# Supplementary material for: Universal Prime Editing Therapeutic Strategy for RyR1-Related Myopathies: A Protective Mutation Rescues Leaky RyR1 Channel
Source: Int J Mol Sci. 2025 Mar 21;26(7):2835. doi: 10.3390/ijms26072835 (PMC11988564; doi:10.3390/ijms26072835)
Supplement: Supplementary file 1 [file ijms-26-02835-s001.zip › ijms-3522865-supplementary.pdf]

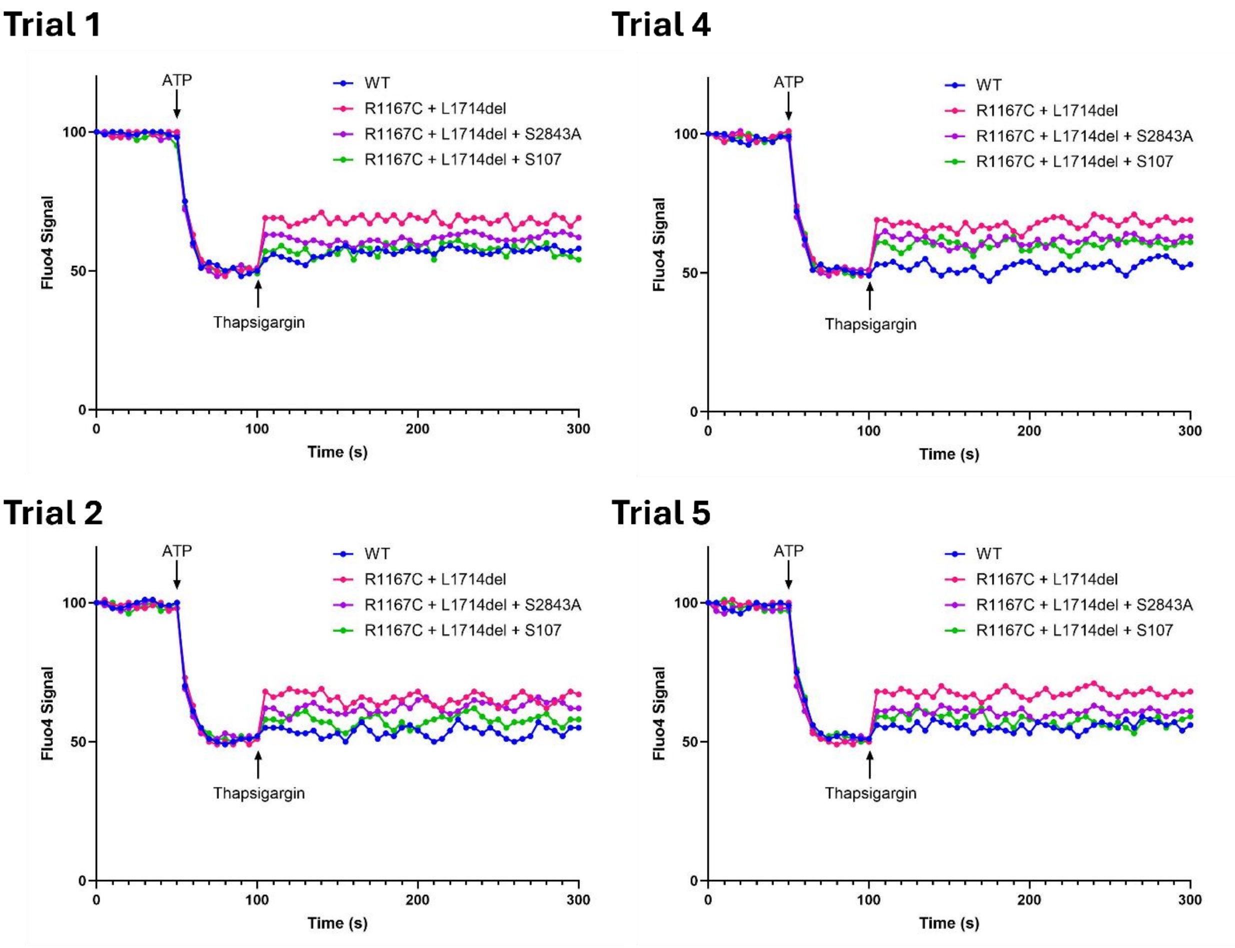

Figure S1. Calcium leak from RyR1 expressing HEK microsomes*. Ca^{2+} leak measured in microsomes from HEK cells expressing WT-RyR1, RyR1-R1667C+1714del, RyR1-R1667C+L1714del+S2843A and RYR1-R1667C+1714del+S107. Five trials of this experiment have been performed (Trial 3 is shown in the main article).*

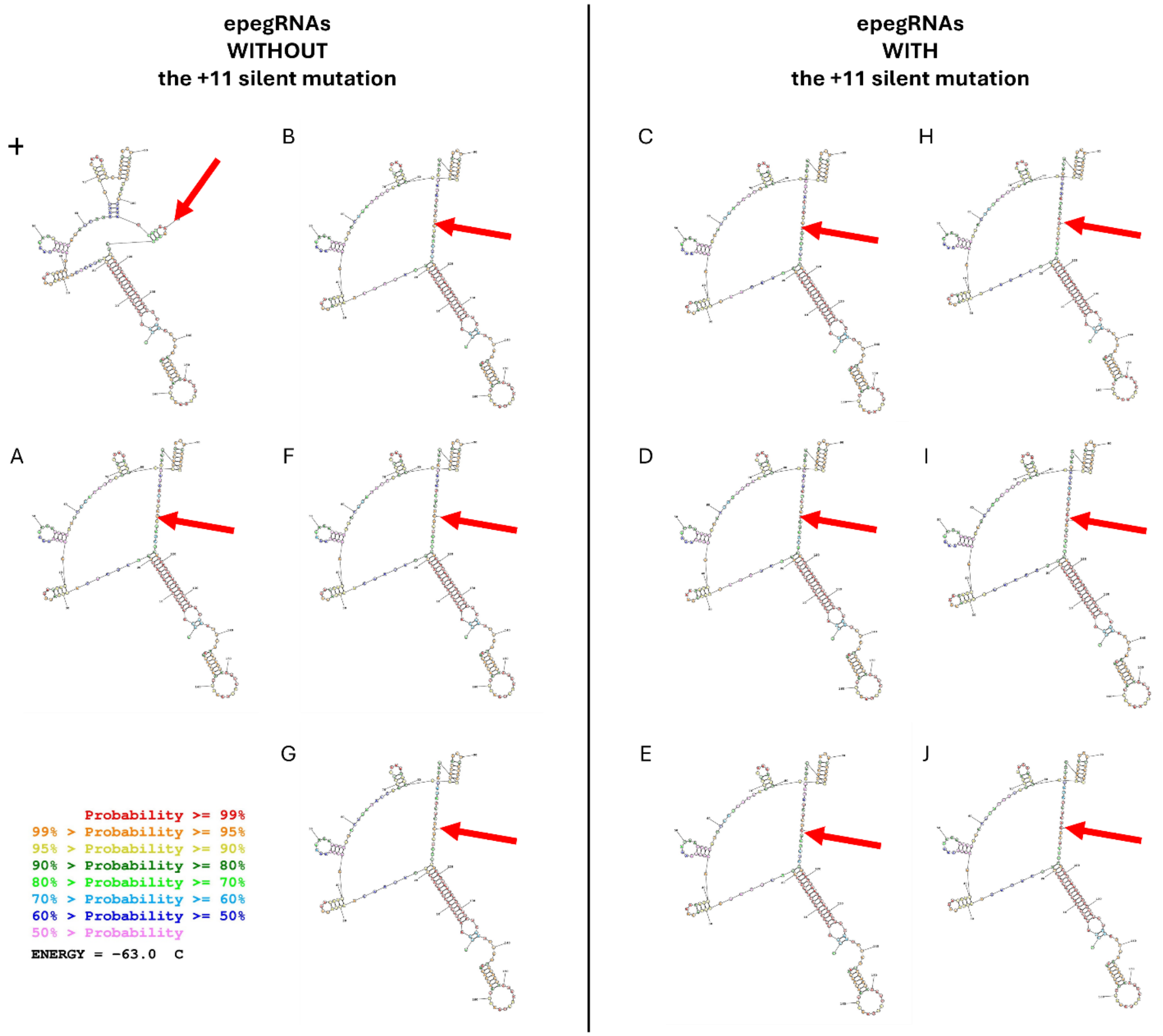

Figure S2. Comparison of epegRNA's structure. *Predicted structures have been produced using the RNAstructure Web Servers for RNA Secondary Structure Prediction, developed by Jessica S. Reuter & Maintained by Richard M. Watson, from Dr David H. Mathews Laboratory. The program https://rna.urmc.rochester.edu/RNAstructureWeb/Servers/Predict1/Predict1.html has been accessed on 2024-09-12. The epegRNAs that do not contain the +11 silent mutation in the RTT can are on the left of the panel. The ones containing the +11 silent mutation in the RTT are on the right of the panel. The red arrows are pointing at the position of the +11 position of the RTT (position 110 of the total epegRNA).*

Table S1. Sequence of the prime editing components and the primers used in this study.

**epegRNA Spacer V1**	
	**Sequence (5'-3')**
**Spacer**	AGACGGAAAAGAAAAAAACG
**Scaffold**	AGAGCTAGAAATAGCAAGTTAAAATAAGGCTAGTCCGTTATCAACTTGAAAAAGTGGCACCGAGTCG
**RTT-PBS_1**	TGCTATCTTCCGCGTTTTTTTTCTTT
**RTT-PBS_2**	TGTGCTATCTTCCGCGTTTTTTTTCTTT
**RTT-PBS_3**	TTTGTGCTATCTTCCGCGTTTTTTTTCTTT
**RTT-PBS_4**	TGCTATCTTCCGCGTTTTTTTTCTTTTCCG
**RTT-PBS_5**	TGTGCTATCTTCCGCGTTTTTTTTCTTTTCCG
**RTT-PBS_6**	TTTGTGCTATCTTCCGCGTTTTTTTTCTTTTCCG
**RTT-PBS_7**	TGCTATCTTCCGCGTTTTTTTTCTTTTCCGTCT
**RTT-PBS_8**	TGTGCTATCTTCCGCGTTTTTTTTCTTTTCCGTCT
**RTT-PBS_9**	TTTGTGCTATCTTCCGCGTTTTTTTTCTTTTCCGTCT
**nsgRNA**	caccgCGTGCCCCTGCCCTCCACCC
**epegRNA Spacer V2**	
	**Sequence (5'-3')**
**Spacer**	GCCTTCACCTGGGCACTTTG
**Scaffold**	AGAGCTAGAAATAGCAAGTTAAAATAAGGCTAGTCCGTTATCAACTTGAAAAAGTGGCACCGAGTCG
**RTT-PBS_1**	AAGATAGCACAAAGTGCCCAGG
**RTT-PBS_2**	GGAAGATAGCACAAAGTGCCCAGG
**RTT-PBS_3**	GCGGAAGATAGCACAAAGTGCCCAGG
**RTT-PBS_4**	AAGATAGCACAAAGTGCCCAGGTGA
**RTT-PBS_5**	GGAAGATAGCACAAAGTGCCCAGGTGA
**RTT-PBS_6**	GCGGAAGATAGCACAAAGTGCCCAGGTGA
**RTT-PBS_7**	AAGATAGCACAAAGTGCCCAGGTGAAGG
**RTT-PBS_8**	GGAAGATAGCACAAAGTGCCCAGGTGAAGG
**RTT-PBS_9**	GCGGAAGATAGCACAAAGTGCCCAGGTGAAGG
**nsgRNA**	TGGACGATAGAGAAGGCCAG
**epegRNA Spacer V3**	
	**Sequence (5'-3')**
**Spacer**	GCCAGGCCCCGCCTTCACCT
**Scaffold**	AGAGCTAGAAATAGCAAGTTAAAATAAGGCTAGTCCGTTATCAACTTGAAAAAGTGGCACCGAGTCG
**PBS**	TGAAGGCGGGGC
**RTT +**	GGAAGATAgCACAAAGTGCTCAGG
**RTT A**	GGAAGATA**g**C**g**CAAAGTGC**T**CAGG
**RTT B**	GGAAGATA**g**C**c**CAAAGTGC**T**CAGG
**RTT C**	GGAAGATA**g**C**g**CA**g**AGTGC**T**CAGG
**RTT D**	GGAAGATA**g**C**t**CA**g**AGTGC**T**CAGG
**RTT E**	GGAAGATA**g**C**c**CA**g**AGTGC**T**CAGG
**RTT F**	GGAAGATA**g**CACAAAG**c**GC**T**CAGG
**RTT G**	GGAAGATA**g**C**t**CAAAG**c**GC**T**CAGG
**RTT H**	GGAAGATA**g**CACA**g**AG**c**GC**T**CAGG
**RTT I**	GGAAGATA**g**C**g**CA**g**AG**c**GC**T**CAGG
**RTT J**	GGAAGATA**g**C**c**CA**g**AG**c**GC**T**CAGG
**nsgRNA**	TGGACGATAGAGAAGGCCA
**epegRNA from IDT**	mC*mC*mC*rArGrGrCrCrCrCrGrCrCrUrUrCrArCrCrUrGrUrUrUrArGrArGrCrUrArGrArArArUrArGrCrArArGrUrUrArArArArUrArArGrGrCrUrArGrUrCrCrGrUrUrArUrCrArArCrUrUrGrArArArArArGrUrGrGrCrArCrCrGrArGrUrCrGrGrUrGrCrGrGrArArGrArUrArGrCrArCrArArArGrUrGrCrCrCrArGrGrUrGrArArGrGrCrGrGrGrGrCrCrGrCrGrGrUrUrCrUrArUrCrUrArGrUrUrArCrGrCrGrUrUrArArArCrCrArArCrUrA*mG*mA*mA
**PCR**	
**Primers**	**Sequence (5'-3')**
PCR F	CAAGGAGTCCCTGAAGGCCA
PCR R	TCCTCTCCATCCCTTCCCTG
Sanger sequencing primer	cttgttctagaccctccccc
